# Isomeric and rotational effects in the chemi-ionisation of 1,2-dibromoethene with metastable neon atoms†

**DOI:** 10.1039/d3fd00172e

**Published:** 2024-02-01

**Authors:** Amit Mishra, Junggil Kim, Sang Kyu Kim, Stefan Willitsch

**Affiliations:** a Department of Chemistry, University of Basel Klingelbergstrasse 80 4056 Basel Switzerland stefan.willitsch@unibas.ch; b Department of Chemistry, KAIST Daejeon 34141 Republic of Korea skkim1230@kaist.ac.kr

## Abstract

The specific geometry of a molecule can have a pronounced influence on its chemical reactivity. However, experimental data on reactions of individual molecular isomers are still sparse because they are often difficult to separate and frequently interconvert into one another under ambient conditions. Here, we employ a novel crossed-beam experiment featuring an electrostatically controlled molecular beam combined with a source for radicals and metastables to spatially separate the *cis* and *trans* stereoisomers as well as individual rotational states of 1,2-dibromoethene and study their specific reactivities in the chemi-ionisation reaction with excited neon atoms. The experiments reveal pronounced isomeric and rotational specificities in the rates and product branching ratios of the reaction. The present study underlines the importance and combined role of molecular geometry and of rotational motion in the dynamics of chemi-ionisation reactions.

## Abstract 2

A new crossed-molecular-beam experiment enabled the study of isomeric and rotational effects in the chemi-ionisation reaction of dibromoethylene with metastable neon atoms.

## Graphical abstract

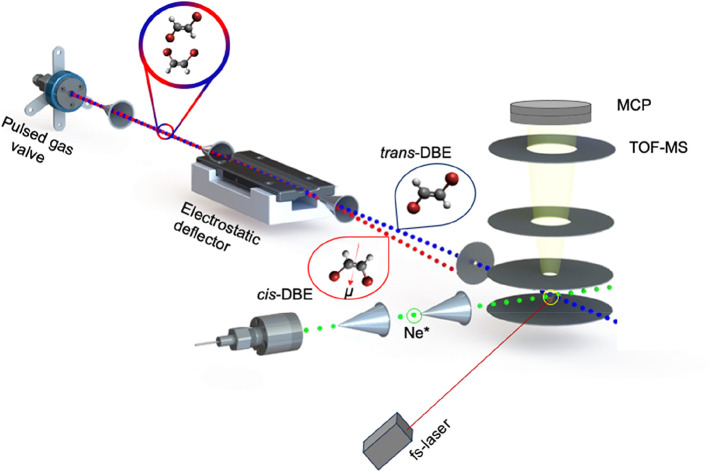

## 1 Introduction

Different spatial orientations of atoms in a molecule lead to different isomers. Subtle structural changes in a molecule can have pronounced effects on its physical and chemical properties resulting in, *e.g.*, different spectral fingerprints,^1^ lifetimes of excited states,^2^ photodissociation dynamics^3^ and chemical reactivities.^4^ Experimental data on reactions of individual molecular isomers, in particular conformers, remain sparse due to the challenges associated with their separation and their tendency to interconvert into one another under ambient conditions. Thus, to enable studies of individual molecular isomers in the gas phase, efficient methods for their separation and preservation under the specific experimental conditions are required. Recent technological advances have led to a markedly improved control over molecules in the gas phase by harnessing electric^5^ and magnetic fields^6,7^ in molecular-beam experiments. In this context, the isolation of isomers based on their different dipole moments using electric fields^8,9^ has proven to be a powerful method for the characterisation of conformational effects in chemical reactions.^10–14^ In these experiments, different conformers were spatially separated from a mixture in a molecular beam by passing them through the inhomogeneous electric field of an electrostatic deflector. The separated isomers were then overlapped with a localised ensemble of cold ions in a trap to study conformer-specific ion–molecule reactions.

Generalising this approach to neutral reactions, we have recently introduced a novel experiment in which an electrostatically deflected molecular beam is crossed at right angles with a beam of metastable atoms for studies of chemi-ionisation (CI) reactions with controlled molecules.^15^ CI reactions play important roles in various contexts including astrochemistry, plasmas and discharge processes.^16–19^ In a CI reaction, a metastable species A* collides with a molecule B resulting in the ejection of an electron and a range of potential products. These can include an adduct AB^+^ (associative ionisation, AI), the ionised molecule B^+^ (Penning ionisation, PI), and various neutral and ionic fragments (dissociative ionisation, DI).

In our apparatus, the electrostatic deflector spatially separates molecules based on their effective dipole moments in the laboratory frame which depend on both the permanent dipole moment in the molecular frame as well as on its rotational state. In previous studies, we have used this approach to select molecules according to either their rotational state^15^ or their conformation^20^ in order to study their respective roles in CI processes. In the present work, we focus on the separation of the geometric, *i.e.*, *cis*/*trans*, isomers of 1,2-dibromoethene (DBE) and the exploration of their distinct reactivities in the CI reaction with metastable Ne* in the (2p)^5^(3s)^1 3^P_2,0_ states.^21^ As the interconversion barrier between the two stereoisomers (estimated to be 1.72 eV at the B3LYP/6-311++G(d,p) level of theory) is high enough to prevent their isomerisation at room temperature, the two isomers can also be separated from a mixture by conventional chromatographic methods for comparison with the *in situ* electrostatic separation in the experiment. This possibility opens up opportunities to disentangle conformational from rotational reactivities, as will be demonstrated here.

## 2 Experimental and theoretical methods

**Fig. 1:**
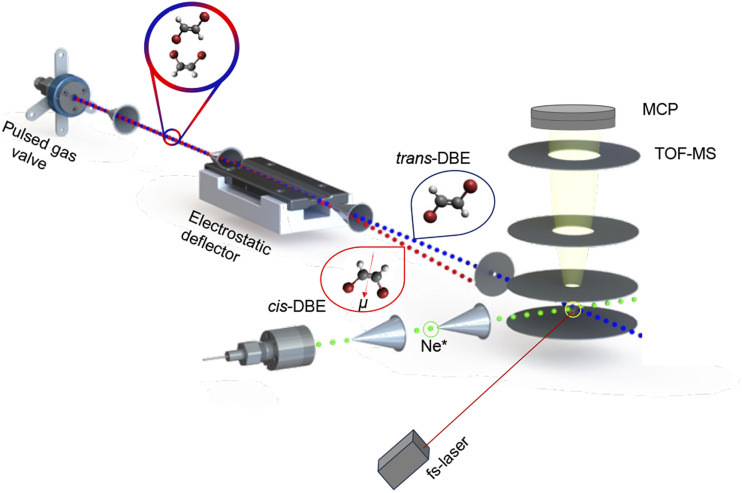
Schematic of the crossed-molecular-beam experiment used in the present study. The polar *cis* and apolar *trans* stereoisomers of 1,2-dibromoethene (DBE) were separated from a mixture using an electrostatic deflector and overlapped with a second beam containing metastable neon atoms (Ne*) in the extraction region of a time-of-flight mass spectrometer (TOF-MS) to study isomer- and rotationally-resolved chemi-ionisation (CI) reactions. See text for details.

A detailed description of this experimental setup can be found in ref. 15. A schematic of the apparatus is presented in Fig. 1. Briefly, a pulsed molecular beam containing a ≈1 : 1 mixture of *cis*- and *trans*-DBE was generated by expanding the vapour pressure from a liquid sample (Sigma-Aldrich, 98%) seeded in helium through an Even–Lavie valve^22^ operated at 40 °C with a backing pressure of 100 bar. Because the energy difference between the two isomers is only ≈30 cm^−1^,^23^ they were approximately equally populated in the beam at the present expansion temperature. For parts of the experiments, the *cis* isomer was separated from the commercial mixture by liquid chromatography and individually entrained in the molecular beam.

The molecular beam was then directed through the inhomogeneous electric field of an electrostatic deflector leading to the spatial separation of the apolar *trans* isomer from the polar *cis* species as illustrated schematically in Fig. 1. The molecular-beam machine was tilted to intersect different parts of the deflected beam with either a femtosecond-laser beam for strong-field multiphoton-ionisation or a beam of metastable neon atoms in the centre of the ion-extraction region of a time-of-flight mass spectrometer (TOF-MS). The ionisation and reaction products were mass-analysed using the TOF-MS. Fs-laser-ionisation and reaction deflection profiles were obtained by integrating specific ion signals in the TOF mass spectra as a function of the tilt of the deflected molecular beam (dubbed the deflection coordinate *y*). The pulsed beam of Ne* was generated by expanding neon gas through a pulsed gas valve at room temperature with a backing pressure of 45 bar by passing through a plate electric-discharge assembly.^24^ From the velocities of the molecular beams colliding at right angles, the collision energy of the reaction was calculated to be 0.36 eV.

The experimental deflection profiles were simulated using a Monte-Carlo trajectory approach as described in detail in ref. 10–12 and 25. For each quantum state of a specific isomer, 50 000 trajectories at different initial conditions were accumulated to generate a simulated deflection profile. The individual state-specific profiles were weighted according to their Boltzmann factors at the rotational temperature of the molecular beam and summed up to yield total profiles for comparison with the experimental data. The dipole moment of *cis*-DBE (*μ* = 1.7 D) required as input for the simulations was calculated using the B3LYP density-functional method and the 6-311++G(d,p) basis set as implemented in Gaussian.^26^

The equilibrium geometries of both isomers in the neutral (S_0_) and cationic (D_0_) ground states were optimised using Møller–Plesset perturbation theory (MP2). From the ground state geometries, adiabatic (AEE) and vertical (VEE) excitation energies as well as transition-dipole moments to electronically excited states were calculated using MP2 and the state-averaged complete-active-space self-consistent-field (SA-CASSCF) method. The active space was comprised of 13 electrons in 12 valence orbitals: six delocalised π/π* orbitals in the molecular plane, four σ/σ* orbitals along the C–Br bonds, and two non-bonding orbitals on each Br atom. The orbitals are depicted in Fig. S1 of the ESI.† The transition energies were further corrected using second-order perturbation theory (CASPT2) with a level shift of 0.4 a.u.^27^ The various dissociation thresholds of the cations of the DBE isomers were calculated from the ionisation energies and optimised energies of the relevant moieties at the MP2 and CASPT2 levels of theory. In these calculations, the aug-cc-pVTZ basis set was used for C and H whereas the aug-cc-pVTZ-PP set was employed for the Br atoms.^28^ All excited-state calculations were carried out using the MOLPRO program package.^29^

## 3 Results

**Fig. 2:**
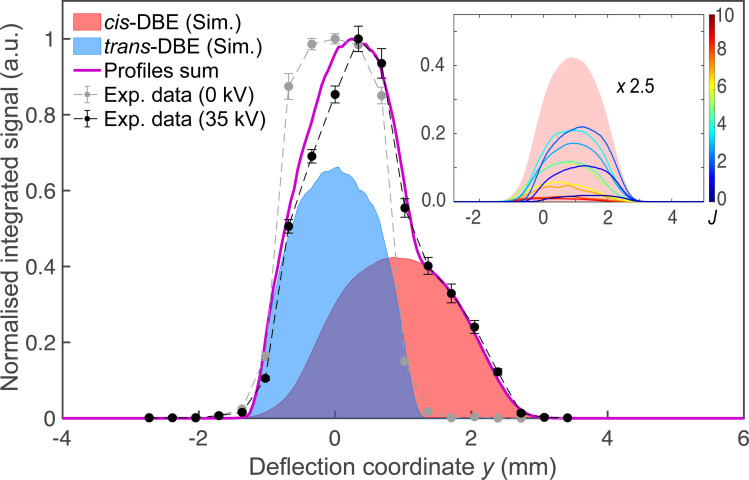
Density profile of the deflected molecular beam of a ≈1 : 1 mixture of *cis*- and *trans*-DBE at deflector voltages of 0 kV (grey symbols) and 35 kV (black symbols) obtained by strong-field multiphoton ionisation in the time-of-flight mass spectrometer and measuring the DBE ion yield as a function of the deflection coordinate *y*. Error bars denote the standard error of three individual measurements consisting of 2000 experimental cycles each. The blue and red areas represent deflection profiles of the *trans*- and *cis*-isomers simulated at a rotational temperature of 1.0 K, the magenta line is their sum. The dashed lines serve as a guide to the eye connecting the experimental data points. Inset: rotational-state resolved deflection profiles of *cis*-DBE and their sum (red-shaded area). The curves are labeled by the rotational quantum number *J* and have been scaled by their relevant Boltzmann factor at the rotational temperature of the beam. Note that states with low *J* are more strongly deflected due to their larger effective dipole moments in the electric field.

Fig. 2 shows a deflection profile of the molecular beam obtained by recording the DBE ion signal in the TOF-MS produced by strong-field fs-laser multiphoton ionisation as a function of the deflection coordinate *y* at a deflector voltage of 0 kV (grey data points) and 35 kV (black data points). The blue and red shaded areas represent simulated deflection profiles for *trans*- and *cis*-DBE, respectively, and the magenta line their sum. The best fit of the simulations to the data was achieved assuming a rotational temperature of 1.0 K for both isomers. While *trans*-DBE possesses no dipole moment and hence is not deflected in the inhomogeneous field, the polar *cis* species shows a clear deflection pattern. The inset in Fig. 2 displays the rotational-state-specific deflection curves of the *cis* isomer weighted by their Boltzmann factors at a rotational temperature of 1.0 K. The deflection profile of each rotational state with quantum number *J* represents a sum of the curves of all relevant asymmetric-top (*τ*) and magnetic (*M*) quantum numbers. It can be seen that the extent of deflection decreases with increasing *J* in line with the polarisability of rotational states in an electric field declining with *J*. Thus, at the highest deflection coordinates the molecular beam is predominantly populated with *cis*-DBE in its lowest rotational states.

**Fig. 3:**
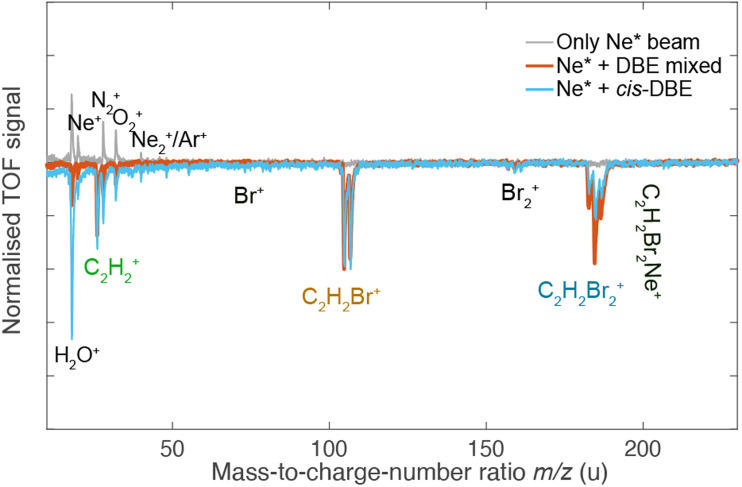
TOF mass spectra of the products of the CI reaction of a ≈1 : 1 mixture of *cis*-/*trans*-DBE (orange trace) and pure *cis*-DBE (blue trace) with Ne*. Intensities are normalised with respect to the C_2_H_2_Br^+^ signal. The main features in the spectrum correspond to ionic products originating from Penning-ionisation (C_2_H_2_Br_2_^+^) and dissociative-ionisation (C_2_H_2_Br^+^, C_2_H_2_^+^) reaction pathways. Grey inverted trace: background experiment conducted without the DBE beam showing the contribution of species arising from CI of background gas in the vacuum chamber and intra-beam Penning ionisation of Ne*.

Fig. 3 shows TOF mass spectra of the products of the CI reaction obtained from overlapping the molecular beams of the mixture of *cis*-/*trans*-DBE (orange trace) and pure *cis*-DBE (blue trace) with Ne* at 0 kV deflector voltage. Three primary product ions appeared at mass-to-charge-number (*m*/*z*) ratios of 186 u, 106 u, and 26 u which were assigned to C_2_H_2_Br_2_^+^, C_2_H_2_Br^+^, and C_2_H_2_^+^. Br^+^ and Br_2_^+^ fragments at *m*/*z* ratios around 80 u and 160 u, respectively, were only barely observed within the sensitivity limits of the experiment. In addition, no AI product NeC_2_H_2_Br_2_^+^ (*m*/*z* = 206 u) was found under the present conditions. The C_2_H_2_Br^+^, C_2_H_2_Br_2_^+^, Br^+^ and Br_2_^+^ peaks show typical splittings according to the natural abundance of the bromine isotopes in these moieties. The remaining features in the spectrum can be attributed to CI of background-gas molecules in the vacuum chamber and intra-beam PI of Ne*, as evidenced by the grey trace which shows a TOF mass spectrum recorded in the absence of the DBE beam.

Comparison of the mass spectra for the mixture and pure *cis*-DBE shows that relative intensity of the PI *vs.* the DI product signals is diminished for the pure *cis* species compared to the mixture. For the mixture of the isomers of DBE, the ratio of PI to DI is determined to be 0.73(4) as derived from the integral of the signal of the PI product and of all DI fragments, while for pure *cis*-DBE the same ratio was obtained to be 0.35(1). This implies that the *cis* isomer preferentially undergoes DI. This finding can be contrasted with the *trans* species for which the same ratio was determined to be 1.45(7) from the data in Fig. 3 implying a higher propensity for PI.

**Fig. 4:**
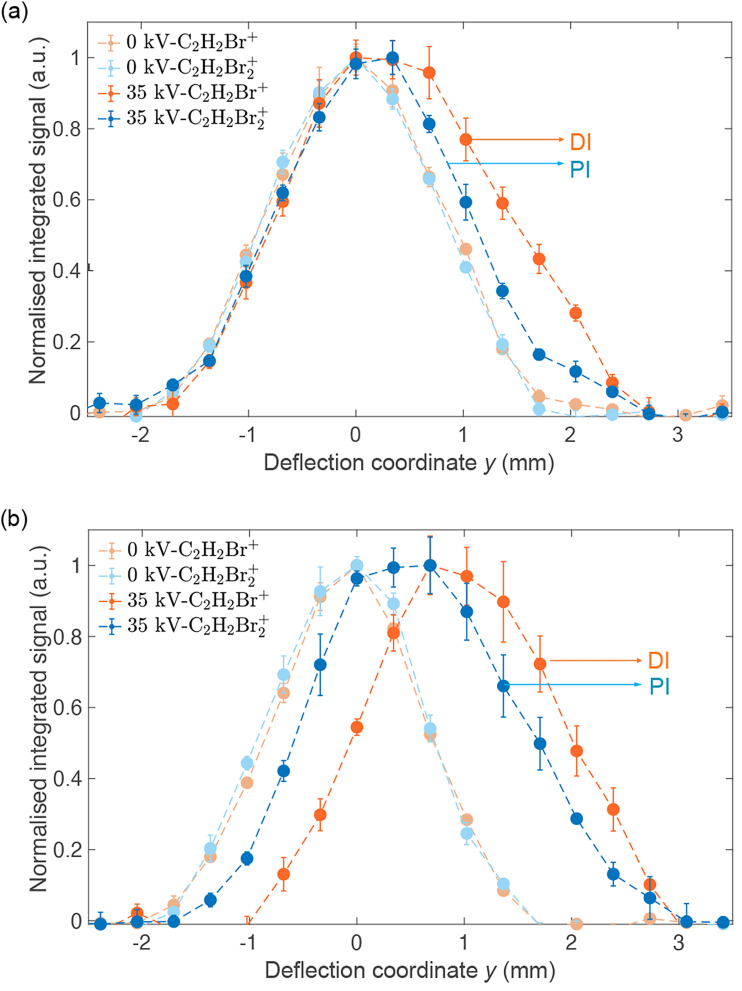
Normalised reaction–deflection profiles of the CI of Ne* with (a) a ≈1 : 1 mixture of *cis*-/*trans*-DBE and (b) pure *cis*-DBE at 0 kV (light symbols) and 35 kV (dark symbols) deflector voltage. Error bars denote the standard error of three individual measurements consisting of 5000 experimental cycles each. The profile of the DI product C_2_H_2_Br^+^ is shifted with respect to the PI product C_2_H_2_Br_2_^+^ suggesting different dynamics for the two reaction channels. See text for details.

Fig. 4(a) shows reaction–deflection profiles of the isomeric mixture of DBE recorded by integrating the C_2_H_2_Br_2_^+^ PI- (blue trace) and C_2_H_2_Br^+^ DI- (red trace) signals in the TOF mass spectrum as a function of the deflection coordinate for deflector voltages of 0 kV (light symbols) and 35 kV (dark symbols). The deflection profile obtained for the C_2_H_2_^+^ fragment has been omitted for clarity as it is identical with the one of C_2_H_2_Br^+^ within the uncertainty limits (Fig. S2 in the ESI shows a comparison of all profiles†). The Br^+^ and Br_2_^+^ fragments were too weak in order to be able to record a reaction–deflection profile with satisfactory signal-to-noise ratio. It can be seen that while the PI- and DI-profiles at 0 kV perfectly overlap, they show appreciable differences at 35 kV. Notably, the profile of the DI fragments extends to larger deflection coordinates compared to the PI product. This is further illustrated in Fig. 4(b) which shows the reaction–deflection profile of pure *cis*-DBE. The reaction–deflection profiles for the PI and DI products are separated from one another with the DI profile extending to larger deflection coordinates.

## 4 Discussion

The findings discussed above can be discussed within the framework of the established mechanism for CI reactions, see, *e.g.*, ref. 16–18 and 30 for a discussion of the most important aspects. At high collision energies, the reaction predominantly proceeds *via* an exchange process in which an electron is transferred from a molecular orbital to the singly occupied valence orbital of the metastable (here one of the three 2p orbitals on Ne). The excited electron is ejected from the metastable and the molecule is left behind in a specific cationic state corresponding to the molecular orbital from which the electron has been removed (PI). If the molecular ion is formed in an excited electronic state above the lowest dissociation limit, the molecule can subsequently fragment leading to DI. Within this orbital picture of CI, the efficiency for the generation of the different cationic states of the molecule is governed by the overlap of the molecular orbital from which the electron is removed with the singly occupied valence orbital on Ne* around the distance of closest approach of the collision partners, *i.e.*, around the turning point of the collision trajectory.

**Fig. 5:**
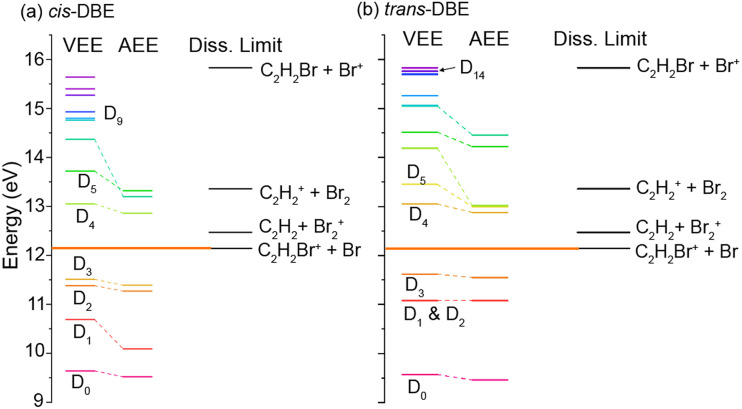
Vertical (VEE) and adiabatic (AEE) excitation energies calculated at the MP2 (ionisation energies) and CASPT2 (excitation energies) levels of theory for different ionic states of (a) *cis*-DBE^+^ and (b) *trans*-DBE^+^ as well as dissociation limits leading to different DI products. Energies are referenced to the S_0_ ground state of the neutral.

Fig. 5 shows the calculated vertical (VEE) and adiabatic (AEE) excitation energies of the electronic states of (a) *cis*- and (b) *trans*-DBE^+^ as well as dissociation energies corresponding to different DI channels. Because electronic excitations occur fast on the time scale of nuclear motions, the VEE are the relevant quantities determining whether a specific ionic state can be reached in the CI process. All energies are referenced to the S_0_ ground state of neutral DBE and states are shown up to an energy of 16 eV. The ionic states D_*i*_ have been labeled in order of ascending VEE from S_0_ (note that this ordering differs from ref. 31 where the states have been ordered according to their VEE from D_0_). The vertical ionisation energy of *cis*-DBE has been calculated to be 9.64 eV at the MP2 level, which can be compared with the experimental values of 9.44 eV and 9. 63 eV in ref. 32 and 38, respectively. The energies are also listed in Tables S1 and S2 of the ESI† together with the dominant electron configurations of the relevant cationic states. The corresponding molecular orbitals are depicted in Fig. S1 while the dissociation energies are listed in Table S3 of the ESI.†

From Fig. 5, it can be seen that the states D_0_–D_3_ lie below the lowest dissociation limit in both isomers. Thus, their population leads to the formation of the PI product C_2_H_2_Br_2_^+^. Each of these states arises from the removal of a single electron from one of the four highest occupied molecular orbitals of DBE, *i.e.*, from one-electron processes (see Table S1†). It can thus be expected that all of these four states are appreciably populated in the CI reaction and yield a significant contribution to the formation of the PI product.

The lowest dissociation threshold corresponding to the C_2_H_2_Br^+^ + Br limit was calculated at 12.14 eV (12.15 eV) for *cis*- (*trans*-)DBE at the MP2 level of theory (orange lines in Fig. 5). Excitations to electronic states above this energy, *i.e.*, to D_4_ and above, can be expected to lead to DI. Indeed, energetically allowed channels resulting in the C_2_H_2_Br^+^ and C_2_H_2_^+^ fragments feature prominently in the experiment (compare Fig. 3), whereas channels resulting in Br_2_^+^ and Br^+^ fragment ions, although also energetically allowed, are only a minor contribution. Although more than ten ionic states of DBE^+^ between the first dissociation threshold and the limiting energy of 16.7 eV of Ne* can in principle be reached in the CI reaction, most of them exhibit electron configurations differing from the S_0_ state by more than one electron (see Tables S1 and S2 of the ESI†). The electronic transition moments from S_0_ to these states can therefore be expected to be small and these transitions are unlikely to strongly contribute to the reaction in a first approximation. Only D_4_, D_5_ as well as D_9_ and D_14_ for *cis*- and *trans*-DBE, respectively, can be reached from S_0_ in a one-electron process implying that excitations to these states dominate the DI dynamics. Overall, the higher yield of DI products for the *cis* species suggests that transitions to states above D_3_ dominate. By contrast, the higher propensity for PI observed for the *trans* isomer points to a predominant population of states below D_4_.

Because the extent of deflection depends on the rotational state (see inset of Fig. 2), the differences between the reaction deflection profiles observed for PI and DI in Fig. 4(b) suggest that *cis*-DBE exhibits different rotational dynamics in these channels. Notably, the larger signal strength of the DI compared to the PI profile at higher deflection coordinates suggests that *cis*-DBE in its lowest rotational states, which are most strongly deflected, appears to be more reactive in DI compared to PI.

A detailed theoretical modelling of the rotational dynamics of PI and DI processes in this system requires knowledge of the relevant optical potentials, *i.e.*, the highly excited potential energy surfaces and decay widths from the collision complex to different final states. However, the accurate calculation of optical potentials is currently out of reach for a system of the present size. In the absence of this information, the origin of the rotational effect observed in the experiment can only be speculated upon. It can be surmised that during the collision of *cis*-DBE with Ne*, the molecule aligns towards the atom along its dipole axis because of dipole-induced dipole interactions.^17,20,33^ Because of the comparatively large dipole moment of *cis*-DBE (*μ* = 1.7 D) and polarisability of Ne* (*α* = 27.8 Å^3^),^34^ this is the leading long-range force in this system. This alignment effect can, in principle, be countered by molecular rotation.^20^ This suggests that in the lowest rotational states, which are the most polarisable and thus the most easy to align, an approach along the dipole axis is favoured and such an approach seems to entail a higher propensity for DI compared to PI.

We note that in this case the collision complex has *C*_2v_ symmetry and the possibilities for PI and DI are restricted as most of the overlap integrals between the relevant orbitals of DBE and Ne* vanish for symmetry reasons. Thus, the decay widths are expected to be negligible for most possible channels. In this framework, the observed rotational effect could be explained if one (or several) of the decay channels entailing the D_4_, D_5_ and D_9_ states dominates over all others in this collision configuration. Detailed calculations would be required to confirm this hypothesis.

It is worth noting that the photofragmentation dynamics of 1,2-DBE were found to be also be quite different for the *cis* and *trans* isomers in the cationic excited states upon picosecond multiphoton excitation over a broad wavelength range, as reported in ref. 31. The observation of a highly anisotropic Br^+^ fragment was ascribed to the D_0_ → D_4_ vertical transition of the *trans* isomer where the transition dipole moment is parallel to the C–Br bond axes. Conversely, as the transition dipole moment is parallel to the C=C bond axis in the D_0_ → D_4_ transition of the *cis* isomer, the observation of a perpendicular transition suggested that the high-kinetic-energy portion of the Br_2_^+^ photofragment should mainly stem from the *cis* species. Thus, the photodissociation dynamics of 1,2-DBE in the cationic excited states has been found to be quite distinct for the two isomers, echoing the present findings for CI.

## 5 Conclusions and outlook

In the present study, we explored the isomer-resolved CI reaction of *cis*- and *trans*-1,2-DBE with metastable neon atoms using a novel crossed-beam experiment which enabled the spatial separation of the two isomers from a mixture as well as of individual rotational states of the *cis* isomer using an electrostatic deflector. PI as well as DI products were observed using time-of-flight mass spectrometry of the generated ions. The results were analysed with the help of quantum-chemical calculations providing information about possible CI mechanisms. Different product branching ratios for PI and DI were observed for the two isomers. Moreover, the DI products appear to be formed with a higher efficiency in low rotational states of *cis*-DBE which was tentatively discussed within the framework of a stereodynamic effect.

The present study (together with ref. 20) introduced a new methodology to explore isomeric and rotational specificities in CI reactions of polyatomic molecules, illustrating the capabilities of controlled-molecular-beam experiments to uncover fine details of these reactions in complex systems. To gain a more comprehensive understanding of the present dynamics, we are currently implementing a velocity-map imaging setup to image the electrons and ionic products of the reaction in coincidence.^35^ This upgrade to the experiment will yield more detailed information on both the ionic electronic states initially populated in the CI process as well as the energy partitioned into the different DI products. Additionally, the preparation of specific rovibrational states of the molecule, facilitated by the present electrostatic separation of rotational states, could provide further insights into rotational and mode-specific dynamics of the present system.

On the theory side, the accurate calculation of optical potentials and the highly excited electronic states involved, which would be vital for a quantitative treatment of the CI dynamics, represent a severe challenge for systems of the complexity and size considered here. The present study thus calls for the development of new methods to treat CI of polyatomic molecules including the effects observed here. While an *ab initio* treatment, as has been achieved for diatomics,^36,37^ may currently be out of reach for systems of the present size, force-field based approaches^30^ may offer a route for further progress.

## Author contributions

A. M. conducted the experiments and analysed the data. J. K. performed the quantum-chemical calculations and assisted in the experiments. S. W. and S. K. K. conceived and supervised the project. All authors contributed to the writing of the manuscript.

## Conflicts of interest

There are no conflicts to declare.

## Supplementary Material

FD-251-D3FD00172E-s001

FD-251-D3FD00172E-s002

FD-251-D3FD00172E-s003
