# Supplementary figures and images for: Isomeric and rotational effects in the chemi-ionisation of 1,2-dibromoethene with metastable neon atoms
Source: Faraday Discuss. 2024 Feb 1;251:92–103. doi: 10.1039/d3fd00172e (PMC11349061; doi:10.1039/d3fd00172e)

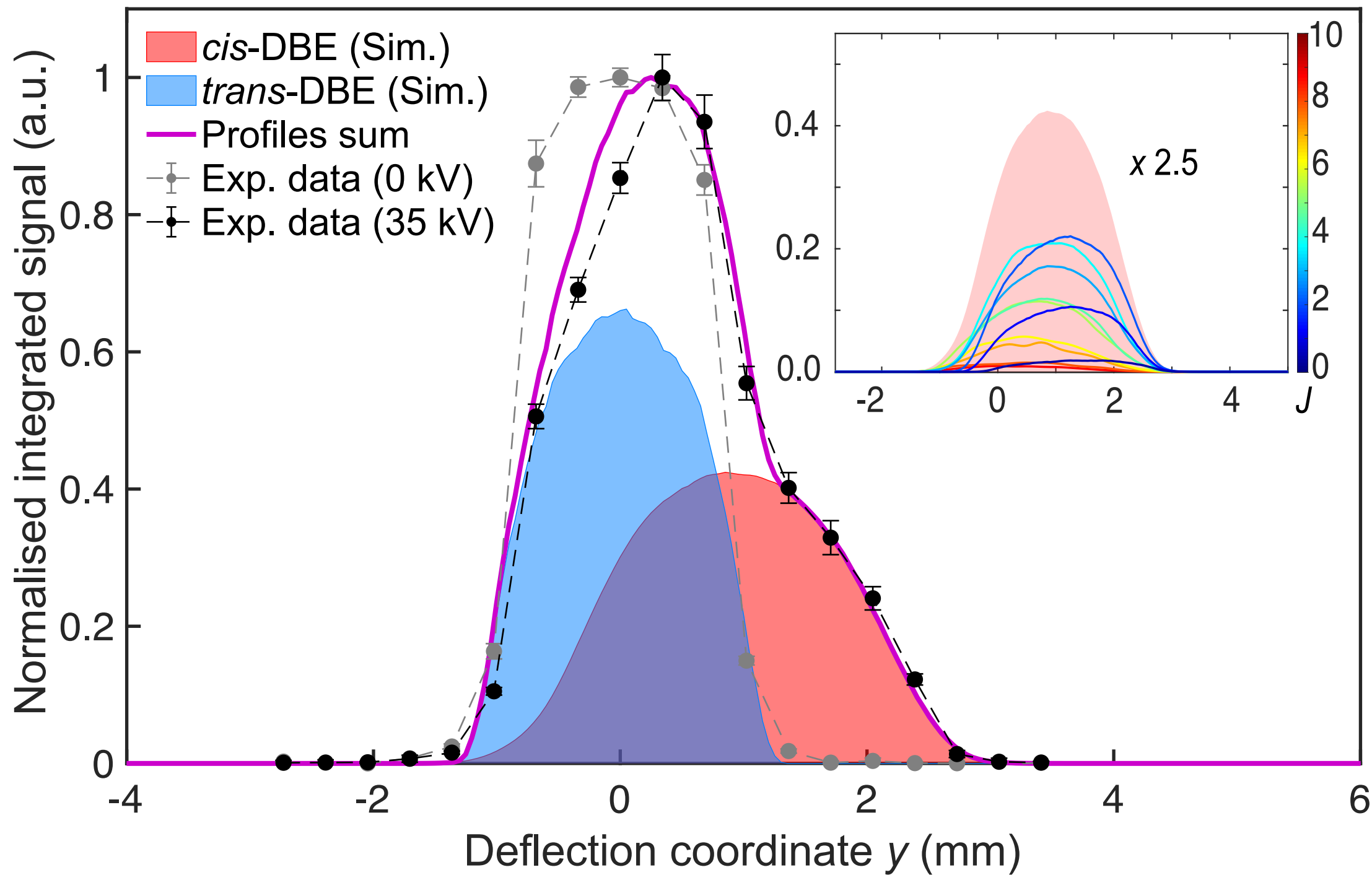

Supplement: FD-251-D3FD00172E-s001 [file FD-251-D3FD00172E-s001.pdf]

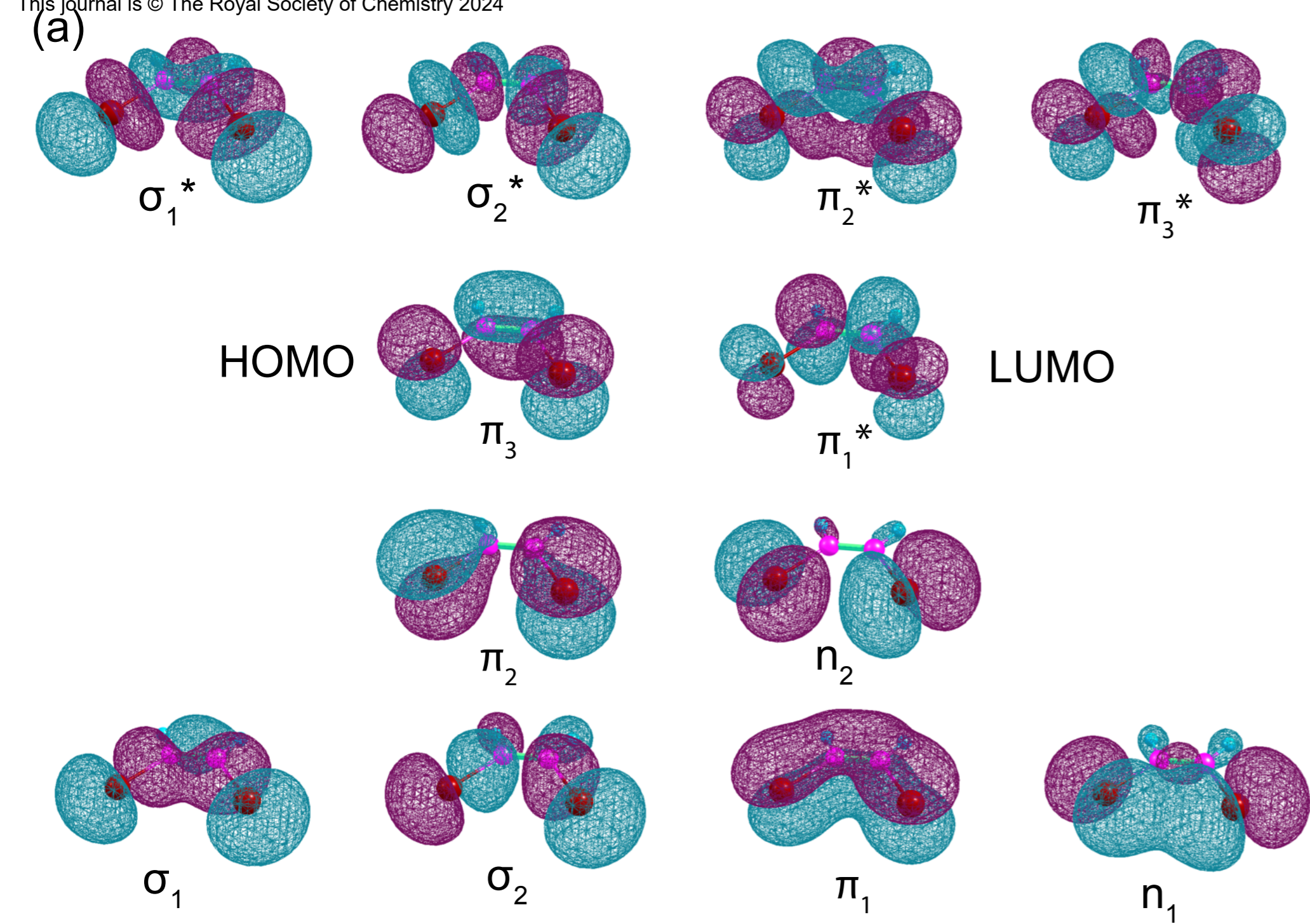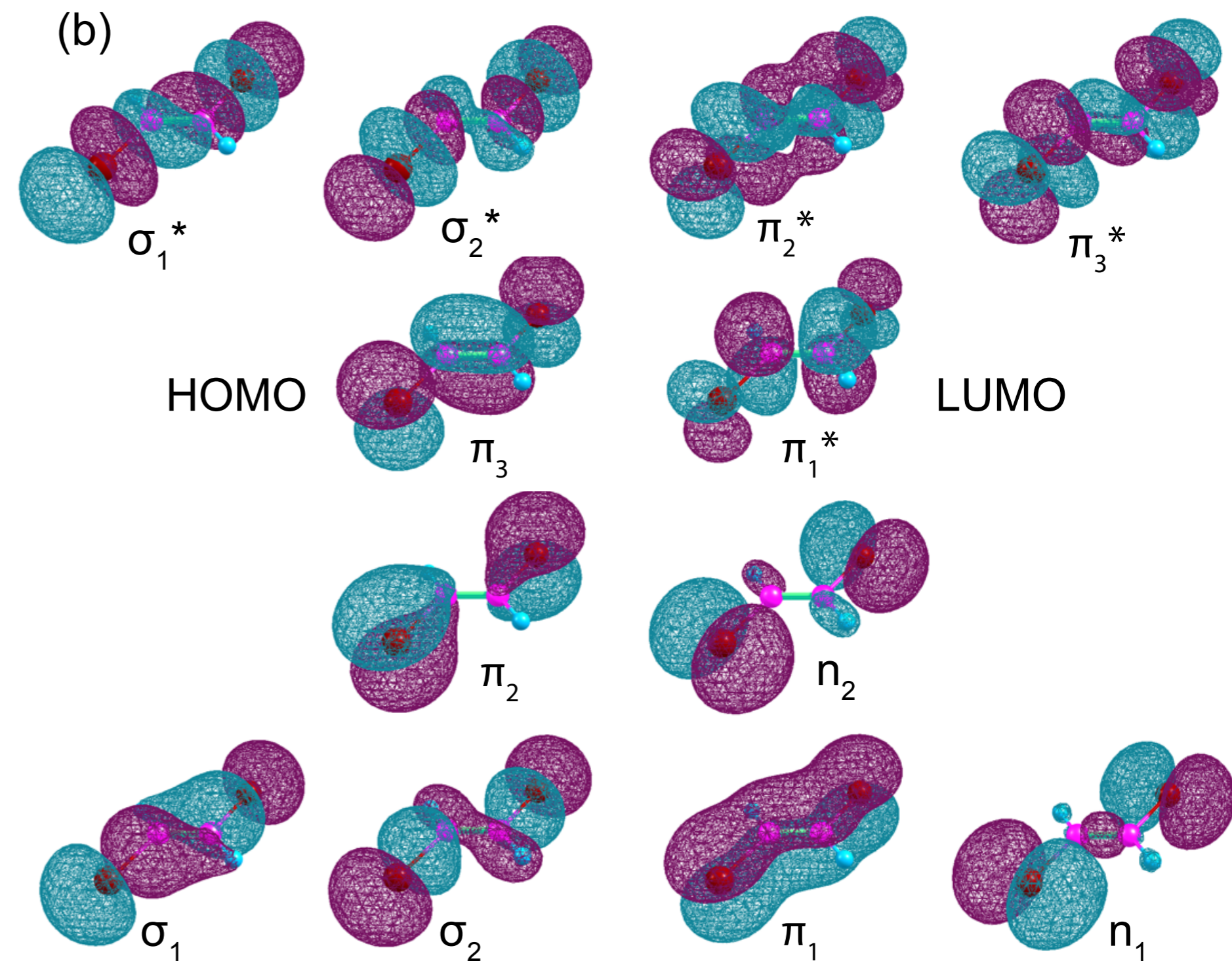

Supplement: FD-251-D3FD00172E-s002 [file FD-251-D3FD00172E-s002.pdf]
