# Supplementary material for: Isomeric and rotational effects in the chemi-ionisation of 1,2-dibromoethene with metastable neon atoms
Source: Faraday Discuss. 2024 Feb 1;251:92–103. doi: 10.1039/d3fd00172e (PMC11349061; doi:10.1039/d3fd00172e)
Supplement: FD-251-D3FD00172E-s003 [file FD-251-D3FD00172E-s003.pdf]

Electronic Supplementary Material (ESI) for Faraday Discussions.
This journal is © The Royal Society of Chemistry 2024

Supplementary Information for

Isomeric and rotational effects in the chemi-ionisation of 1,2-dibromoethene with metastable neon atoms

Amit Mishra,[a] Junggil Kim,[b] Sang Kyu Kim,[*b], and Stefan Willitsch[*a]

[a] *Department of Chemistry, University of Basel, Klingelbergstrasse 80, 4056, Basel, Switzerland.*
[b] *Department of Chemistry, KAIST, Daejeon 34141, Republic of Korea.*
[*] *E-mail: stefan.willitsch@unibas.ch (S.W.), skkim1230@kaist.ac.kr (S.K.K.)*

State	Symmetry	Configuration	MP2	CASPT2	Expt. [32]	Expt. [38]
D_0	$^2B_1$	$(\pi_3)^{-1}$	9.64	9.34	9.44	9.63
D_1	$^2B_2$	$(n_2)^{-1}$	10.69	10.39	10.74	10.74
D_2	$^2A_1$	$(n_1)^{-1}$	11.38	11.08	11.24	11.23
D_3	$^2A_2$	$(\pi_2)^{-1}$	11.51	11.21	11.56	11.53
D_4	$^2B_1$	$(\pi_1)^{-1}$	13.05	12.75	12.85	12.86
D_5	$^2B_2$	$(\sigma_2)^{-1}$	13.72	13.42	13.27	13.22
D_6	$^2A_2$	$(\pi_3)^{-2}(\pi_1^*)^1$	14.37	14.07	-	-
D_7	$^2A_1$	$(n_2)^{-1}(\pi_3)^{-1}(\pi_1^*)^1$	14.76	14.46	-	-
D_8	$^2A_1$	$(\sigma_2)^{-1}(\pi_3)^{-1}(\sigma_1^*)^1$	14.80	14.50	-	-
D_9	$^2A_1$	$(\sigma_1)^{-1}$	14.93	14.63	14.80	14.83
D_{10}	$^2A_2$	$(n_2)^{-1}(\pi_3)^{-1}(\sigma_1^*)^1$	15.27	14.98	-	-
D_{11}	$^2B2$	$(n_1)^{-1}(\pi_3)^{-1}(\pi_1^*)^1$	15.40	15.11	-	-
D_{12}	$^2B1$	$(n_1)^{-1}(\pi_3)^{-1}(\sigma_1^*)^1$	15.64	15.34	-	-

Table S1: Vertical excitation energies (VEE) in eV and dominant electron configurations of electronically excited states of *cis*-DBE^+. The VEEs were calculated at the MP2 and CASPT2 levels of theory and are compared with experimental results. All energies are referenced to the S_0 electronic ground state of the neutral.

State	Symmetry	Configuration	MP2	CASPT2	Expt. [32]	Expt. [38]
D_0	^2A_u	$(\pi_3)^{-1}$	9.57	9.22	9.44	9.55
D_1	^2A_g	$(n_2)^{-1}$	11.08	10.73	11.05	11.04
D_2	^2B_u	$(n_1)^{-1}$	11.08	10.73	11.05	11.04
D_3	^2B_g	$(\pi_2)^{-1}$	11.62	11.27	11.60	11.57
D_4	^2A_u	$(\pi_1)^{-1}$	13.05	12.69	13.0	12.90
D_5	^2A_g	$(\sigma_2)^{-1}$	13.45	13.10	13.00	13.30
D_6	^2B_g	$(\pi_3)^{-2}(\pi_1^*)^1$	14.19	13.83	-	-
D_7	^2B_u	$(\pi_3)^{-2}(\sigma_1^*)^1$	14.52	14.17	-	-
D_8	^2A_g	$(n_1)^{-1}(\pi_3)^{-1}(\pi_1^*)^1$	15.05	14.70	-	-
D_9	^2B_u	$(n_2)^{-1}(\pi_3)^{-1}(\pi_1^*)^1$	15.06	14.70	-	-
D_{10}	^2B_g	$(n_2)^{-1}(\pi_3)^{-1}(\sigma_1^*)^1$	15.26	14.90	-	-
D_{11}	^2A_u	$(n_1)^{-1}(\pi_3)^{-1}(\sigma_1^*)^1$	15.26	14.91	-	-
D_{12}	^2A_u	mixed	15.69	15.34	-	-
D_{13}	^2B_g	mixed	15.70	15.35	-	-
D_{14}	^2B_u	$(\sigma_1)^{-1}$	15.76	15.41	15.90	15.50
D_{15}	^2A_g	mixed	15.83	15.48	-	-

Table S2: Vertical excitation energies (VEE, in eV) and dominant electron configurations of electronically excited states of *trans*-DBE^+. The VEEs were calculated at the MP2 and CASPT2 levels of theory and are compared with experimental results. All energies are referenced to the S_0 electronic ground state of the neutral.

	cis-DBE		*trans*-DBE	
Product channel	MP2	CASPT2	MP2	CASPT2
C_2H_2 + Br_2	1.89	1.86	1.87	1.80
C_2H_2 + Br + Br	4.18	3.98	4.16	3.92
C_2H_2Br + Br	4.20	3.62	4.12	3.48
$C_2H_2Br_2^+$	9.52	9.22	9.46	9.11
$C_2H_2Br^+$ + Br	12.15	11.98	12.14	11.91
C_2H_2 + Br_2^+	12.48	12.15	12.47	12.08
$C_2H_2^+$ + Br_2	13.37	13.10	13.35	13.04
$C_2H_2^+$ + Br + Br	15.67	15.23	15.65	15.17
C_2H_2 + Br + Br^+	15.84	15.44	15.82	15.38
C_2H_2Br + Br^+	15.86	15.08	15.79	14.94

Table S3: Calculated appearance energies (AE) of different dissociation products. All energies are referenced to the S_0 electronic ground state of the neutral.

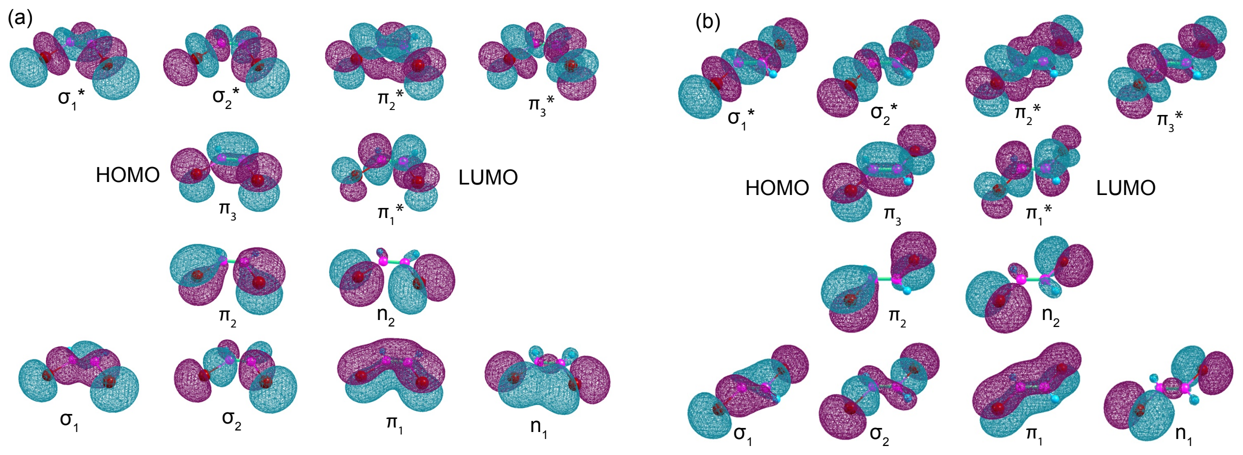

Figure S1: Relevant molecular orbitals of (a) *cis*- and (b) *trans*-dibromoethene calculated at the CASSCF level of theory.

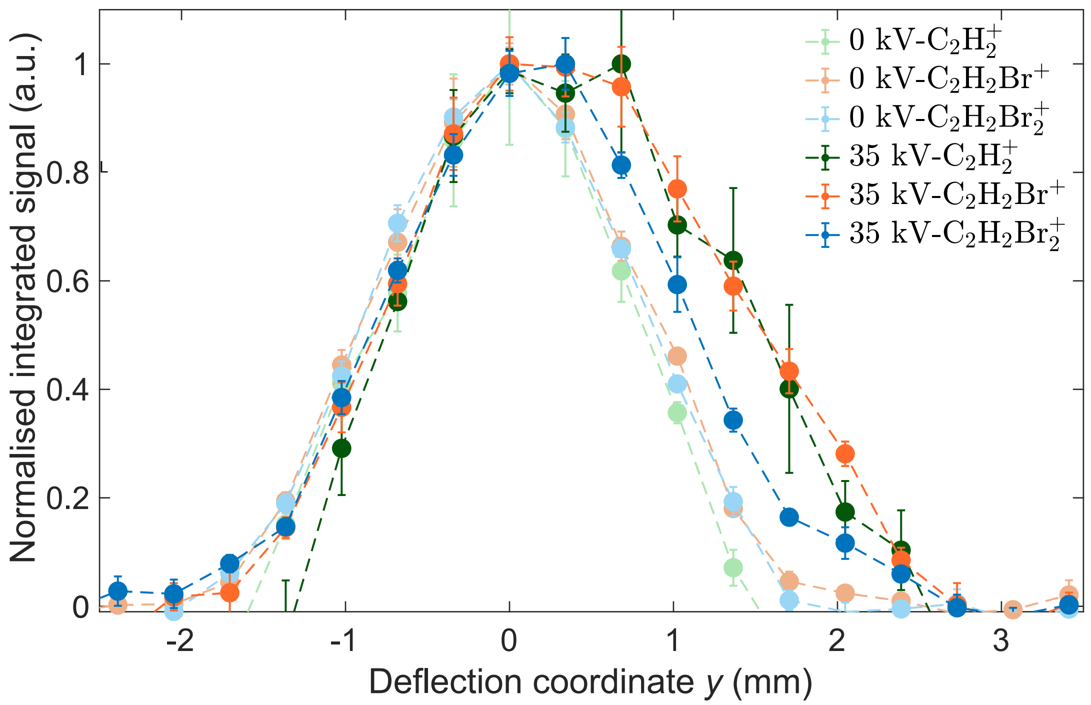

Figure S2: Normalised deflection profiles of the chemiionisation reaction of a 1:1 mixture of *cis-/trans*-dibromoethene with Ne* recorded for Penning-ionisation (PI, blue) and dissociative-ionisation (DI, green and orange) products at 0 kV (light symbols) and 35 kV (dark symbols) deflector voltages.
